# Robot-Assisted Cystectomy With Intracorporeal Diversion for Bladder Intra-diverticular Tumor: A Special Case Report

**DOI:** 10.7759/cureus.81019

**Published:** 2025-03-22

**Authors:** Doan H Pham, Cam Hoang Nguyen Phuc, Chuyen L Vu, Phuong V Do

**Affiliations:** 1 Department of Urology, Binh Dan Hospital, Ho Chi Minh City, VNM; 2 Department of General Surgery, Pham Ngoc Thach University of Medicine, Ho Chi Minh City, VNM; 3 Department of Urology and Andrology, Pham Ngoc Thach University of Medicine, Ho Chi Minh City, VNM; 4 Department of Urology, Tam Anh Hospital, Ho Chi Minh City, VNM

**Keywords:** gross hematuria, intracorporeal urinary diversion, intra-diverticular bladder tumor, papillary transitional cell carcinoma, robot-assisted radical cystectomy

## Abstract

This report details the case of a 60-year-old male with a grade 3 intra-diverticular bladder tumor originating from an acquired bladder diverticula. The patient presented with painless gross hematuria lasting three weeks. Imaging and histopathological analysis confirmed a high-grade papillary transitional cell carcinoma. He underwent robot-assisted radical cystectomy with pelvic lymphadenectomy and the creation of an intracorporeal Studer neobladder. The surgery was successful, and the patient had an uneventful recovery. Intra-diverticular bladder tumors are rare and pose diagnostic and therapeutic challenges due to their unique anatomy and higher grade. Robot-assisted radical cystectomy has become the preferred approach at high-volume centers, providing reduced morbidity and excellent outcomes. For advanced cases, multimodal treatments such as neoadjuvant chemotherapy are crucial, emphasizing the need for individualized care. This case highlights the effectiveness of personalized, minimally invasive techniques and the importance of multidisciplinary collaboration in managing bladder tumors within diverticula.

## Introduction

Bladder diverticula vary in size, with dimensions ranging from 10 cm, though the average size is approximately 5 cm [1]. Typically, they are characterized by thin walls and a narrow neck or ostium that communicates with the bladder lumen [2]. In adults, bladder diverticula are predominantly acquired rather than congenital in origin.

Due to the association between bladder diverticula and urinary stasis, there is an elevated risk of complications such as urolithiasis, chronic inflammation, and recurrent infections. The incidence of neoplasms in patients with bladder diverticula is higher than in the general bladder population, with reported rates ranging from 0.8% to 13%. Among these neoplasms, intra-diverticular bladder tumors represent 1% of all bladder cancers, and they occur more frequently in males, with a male-to-female ratio of 9:1 [3].

For patients presenting with high-grade intra-diverticular bladder tumors, particularly those with concomitant high-grade cancer elsewhere in the bladder, radical cystectomy is generally recommended. Robot-assisted radical cystectomy (RARC) has proven to be a safe and effective procedure, yielding oncologic outcomes comparable to conventional open radical cystectomy (ORC). Furthermore, intracorporeal urinary diversion (ICUD) is increasingly being incorporated into RARC [4]. At high-volume centers, robot-assisted approaches to diverticulectomy and cystectomy have become the preferred treatment modality.

This report highlights the case of a 60-year-old male diagnosed with a grade 3 intra-diverticular tumor arising from an acquired bladder diverticulum, successfully treated with robot-assisted radical cystectomy.

## Case presentation

A 60-year-old male was admitted to our Urology Department with a 3-week history of painless gross hematuria, accompanied by blood clots. On physical examination, the patient’s vital signs were stable, and no other symptoms such as dysuria, fever, or weight loss were noted. The patient had previously undergone a Transurethral Resection of the Prostate (TURP) three years earlier and had a history of hypertension.

**Figure 1:**
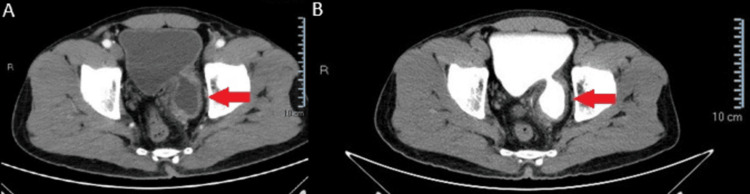
CT scan images without contrast (A) and with contrast (B) of bladder diverticulum (red arrow)

The differential diagnoses included bladder cancer and lower urinary tract infection. Blood tests revealed normal results, while urine analysis showed the presence of red blood cells. A computed tomography (CT) scan identified a bladder diverticulum measuring 54 × 48 mm with an uneven wall (maximum diameter 13 mm) on the left posterior wall of the bladder. Additionally, there was thickening of the bladder dome (8 mm) and invasion of surrounding fat, with no regional lymph node involvement (Figure 1).

Following the initial cystoscopy, multiple sizable papillary bladder tumors were identified within a large bladder diverticulum. The diverticulum was located near the bladder neck, extending towards the posterior region of the bladder, beneath and adjacent to the left ureteric orifice, with dimensions of approximately 6 × 5 × 4 cm. The bladder tumors were observed around the circumference of the diverticulum. A partial resection was performed using transurethral resection of the bladder tumor (TURBT), and tumor specimens were sent for histological analysis. The histology revealed a diagnosis of grade 3 papillary transitional cell carcinoma (TCC).

**Figure 2:**
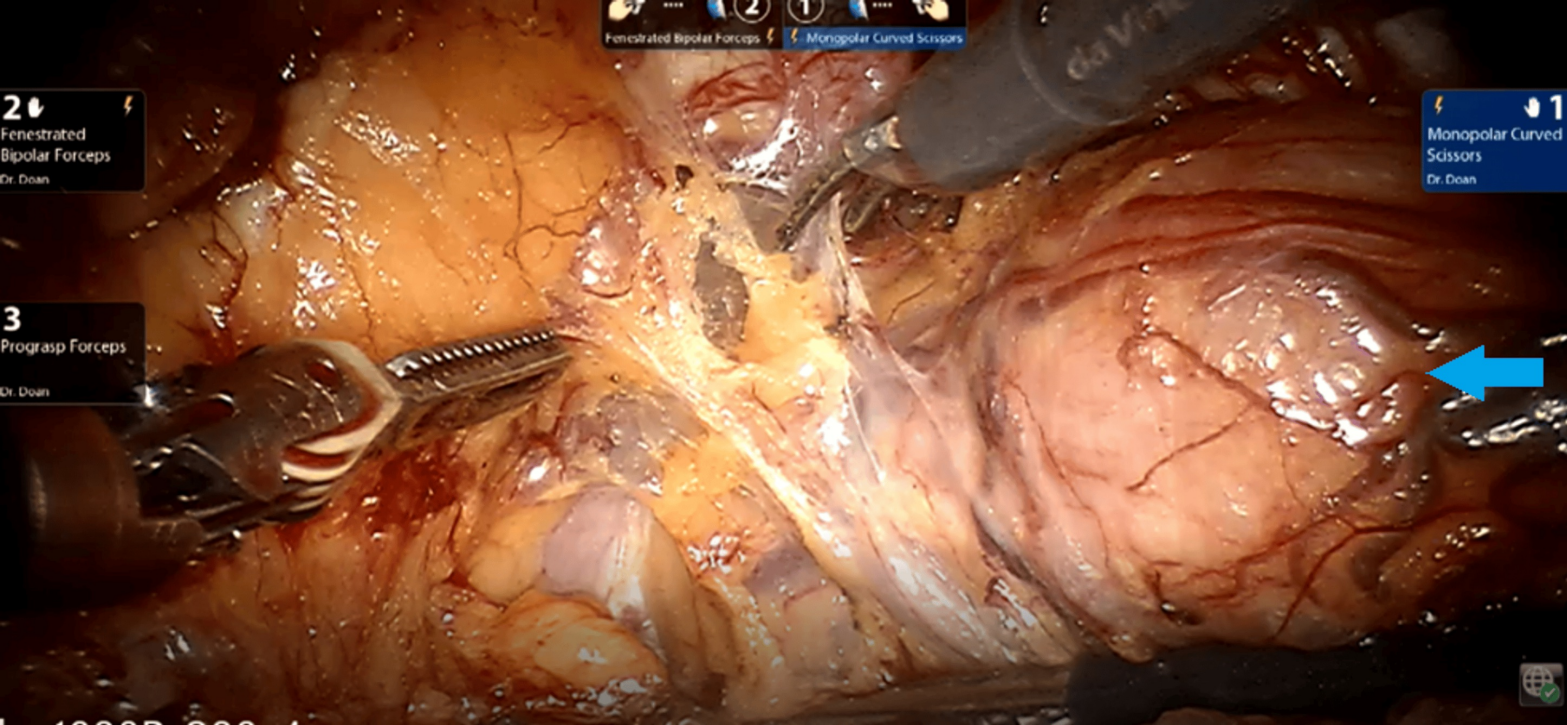
Robot-assisted radical cystectomy Blue arrow: Intra-diverticular bladder tumor

Based on these findings, the medical team recommended surgical intervention. The patient underwent robot-assisted radical cystectomy with pelvic lymphadenectomy and intracorporeal neobladder (Studer pouch) reconstruction (Figure 2). A critical aspect of the procedure was achieving a tension-free ureteroileal anastomosis, which posed a technical challenge. The console time was 390 minutes, with an estimated blood loss of 500 ml. The procedure was completed without intraoperative or postoperative complications. The patient resumed a regular diet on postoperative day 1 and was discharged on postoperative day 8.

**Figure 3:**
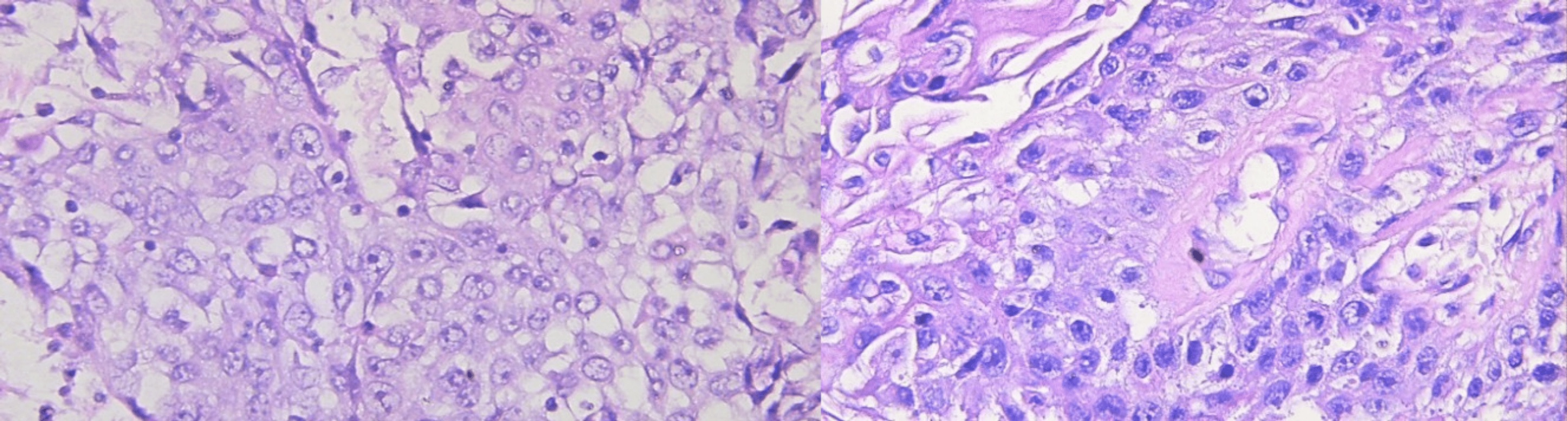
Pathology results of high-grade transitional cell carcinoma

Pathological examination confirmed that the bladder diverticulum had an acquired origin, as evidenced by the absence of muscularis propria. Biopsies taken from the tumor mass within the diverticulum identified it as a high-grade transitional cell carcinoma (TCC), with invasion into the serosa (T3b) (Figure 3). Additional biopsies from the primary bladder mass revealed high-grade TCC, infiltrating the subepithelial connective tissue (T1), and involvement of one regional lymph node (N1). Notably, the surgical resection margins, including those of the prostate and left ureter, were free of neoplastic cells.

## Discussion

Bladder diverticula are defined as herniations of the bladder mucosa through areas of weakness in the detrusor muscle. Intra-diverticular bladder tumors, first described by Targett in 1896, are a rare occurrence, accounting for only 1% of all bladder tumors [5]. These tumors predominantly arise in acquired diverticula (often multiple lesions) and are more commonly observed in adults. In contrast, congenital diverticula are typically solitary, located near the uretero-vesical junction, and rarely present in adults [6].

The primary manifestation of bladder transitional cell carcinoma (TCC), regardless of location, is painless hematuria, which is also characteristic of bladder diverticula. The structural features of diverticula, such as their thin walls and absence of a muscularis propria layer, predispose them to complications, including urinary stasis, infection, and malignancy. These factors also contribute to the diagnostic and therapeutic challenges posed by intra-diverticular bladder tumors.

Histologically, urothelial carcinoma is the most common malignancy found in bladder diverticula (78%), followed by squamous cell carcinoma (17%), mixed urothelial and squamous cell carcinoma (2%), and adenocarcinoma (2%). Compared to tumors located in the main bladder, intra-diverticular bladder tumors tend to be of a higher grade and are often asymptomatic, further complicating diagnosis and treatment. Standard bladder cancer staging systems are not directly applicable to diverticular tumors due to their unique anatomical characteristics. The staging of bladder tumors generally includes non-invasive (Tis/Ta), superficially invasive (T1), extra-vesical (T3), and tumors invading adjacent structures (T4) [7].

The diagnostic workup for intra-diverticular bladder tumors is largely similar to that for primary bladder cancer, encompassing urine cytology, cystoscopy, imaging, and pathological examination. Urine cytology is non-invasive and highly specific, but its sensitivity is limited, particularly for low-grade tumors [7]. Cystoscopy, an essential diagnostic tool, offers direct visualization of the bladder cavity; however, narrow diverticular necks may hinder tumor detection [8]. Imaging modalities such as ultrasound (US) and computed tomography (CT) are commonly employed, with CT providing superior diagnostic and staging accuracy. Biopsy remains critical to confirm malignancy.

Treatment options for intra-diverticular bladder tumors range from transurethral resection (TUR) to radical cystectomy. TUR is effective for non-invasive tumors (stage Ta) but carries the risk of bladder perforation, particularly in thin-walled diverticula [8]. Partial cystectomy or diverticulectomy may be considered when TUR is inadequate, while radical cystectomy is indicated for advanced-stage tumors [7]. Although radical cystectomy is highly effective, it is associated with significant morbidity and necessitates urinary diversion, which can impact patients’ quality of life [7]. Studies show that outcomes are improved in high-volume centers, where surgeons perform ≥8 radical cystectomies annually, achieving lower mortality and morbidity rates [9,10].

Robot-assisted radical cystectomy (RARC) with intracorporeal urinary diversion (ICUD) is increasingly recognized as a preferred approach, particularly in high-volume centers. Despite its technical complexity, RARC offers several advantages, including reduced rates of distal ureteral ischemia and intraoperative blood transfusion, leading to faster recovery. Ongoing prospective, randomized clinical trials are comparing RARC to conventional open radical cystectomy (ORC) to further elucidate its benefits [4].

Multimodal therapy integrating systemic treatments such as chemotherapy with surgery is gaining prominence in the management of high-grade tumors. Neoadjuvant chemotherapy, in particular, may improve survival and surgical outcomes for appropriately selected patients with advanced bladder tumors in diverticula. Treatment plans should be individualized, considering the tumor’s anatomical characteristics, the patient’s overall health, and input from a multidisciplinary team. Clinical understaging remains a significant concern, with up to 55% of Ta and T1 cases being understaged following radical cystectomy, underscoring the importance of thorough preoperative assessment [7,8]. Robot-assisted techniques are increasingly favored due to their ability to minimize surgical morbidity while ensuring optimal oncological outcomes. Patients are encouraged to seek care at high-volume centers to benefit from specialized expertise and advanced technologies.

## Conclusions

The management of bladder tumors occurring within diverticula is evolving. The traditional approach of radical surgery for all cases is being replaced by a more personalized and selective treatment strategy tailored to the specific needs of each patient. Given the variety of procedures required for treatment, a robot-assisted approach has become increasingly favorable. When feasible, robotics can help minimize surgical morbidity while optimizing patient outcomes. For the best results, patients diagnosed with this condition should seek treatment at a high-volume center with specialized expertise.
